# Snuffbox Versus Wrist Radiocephalic Arteriovenous Fistulas: 10 Years Experience

**DOI:** 10.7759/cureus.57442

**Published:** 2024-04-02

**Authors:** Mohamed S M Elshikhawoda, Aidas Raudonaitis, Tarig Barakat, Sohaib Jararaa, Mahmoud Okaz, Ebimobo Tobias Keme, Abdillahi Ahmed Roble, Waseem Ahmad, Sherif A Mansour, Ahmed Elmallah

**Affiliations:** 1 Vascular Surgery, Glan Clwyd Hospital, Rhyl, GBR; 2 Vascular and Endovascular Surgery, Glan Clwyd Hospital, Rhyl, GBR

**Keywords:** snuffbox arteriovenous fistula, hemodialysis vascular access, renal access, wrist arteriovenous fistula, arteriovenous fistula

## Abstract

Background

The wrist radiocephalic arteriovenous fistula (RCAVF) is the initial suggested procedure for establishing hemodialysis vascular access (HVA) in the most distal site of the upper limb. The anatomical snuffbox arteriovenous fistula (SBAVF) is barely utilised, despite its remote location. In this study, we aimed to analyse and compare the results of SBAVF and RCAVF in terms of their maturity, patency, and failure rates.

Methodology

This descriptive, retrospective study compared outcomes between SBAVF and RCAVF in terms of maturation, patency, and failure. All patients with chronic kidney disease who attended and underwent either procedure at Betsi Cadwaladr University Health Board between 2013 and 2023 were studied.

Results

In a period of 10 years, 179 patients were included. Overall, 102 (57%) were male and 77 (43%) were female, with a male-to-female ratio of 1.3:1. Wrist radiocephalic fistula was the dominant type of surgery done in 76% (n = 136), while the snuffbox radiocephalic fistula was done in fewer than 24% (n = 43) of patients. Most patients underwent a successful arteriovenous (AV) fistula (n = 105, 58.7%), in contrast to 67 patients whose fistulas failed. There was a significant relationship between fistula failure and complications (p = 0.000). There was no significant effect of the fistula site, hypertension, diabetes, cardiac diseases, smoking, peripheral vascular disease, or central vein stenosis on the failure of the AV fistula (p = 0.127, 0.534, 0.510, 0.397, 0.017, 0.68, and 0.371, respectively).

Conclusions

The snuffbox AV fistula is a suitable and feasible first choice for patients on hemodynamic therapy.

## Introduction

Chronic kidney disease (CKD) is a global public health challenge, and the number of patients with end-stage renal disease (ESRD) has been increasing globally through the years. In Europe, patients with CKD account for 10% of the population. The dialysis population is growing at a rate of about 2% each year [1]. Despite the advances in kidney transplantation, hemodialysis remains the most widely used modality of treatment for patients with ESRD [2,3].

Native arteriovenous fistula (AVF) is the definitive haemodialysis vascular access (HVA) used for chronic haemodialysis. According to guidelines, AVF should be end-to-side radiocephalic (RCAVF), as the most distal site, in the non-dominant upper limb [4].

The anatomical snuffbox AVF (SBAVF) was first described by Rassat et al. in 1969. Despite being more distal compared to RCAVF, it is not frequently used in the United Kingdom [5].

The Fistula First Initiative advocates for the insertion of autogenous haemodialysis access before utilising a prosthetic graft [4,6].

At our centre, SBAVF is considered a first choice for AVF. It is hypothesized that this would spare RCAVF as a second option if it is used as HVA for a period and occluded eventually. In addition, it may reduce the maturity time for future RCAVFs. On the other hand, when occluded, the relatively close wrist cephalic vein may become unusable, which might affect haemodialysis patients negatively until a definitive native HVA is achieved.

This study aims to assess the access outcomes, complications, primary, primary assisted, and secondary patency rates of SBAVF and RCAVF and determine if SBAVF should be the first recommended HVA (in suitable patients) or if it has its drawbacks.

## Materials and methods

Study design and population

This descriptive, observational, and retrospective study compared the outcomes between the snuffbox and wrist arteriovenous (AV) fistula in terms of maturation, patency, and failure. All patients with CKD who attended and underwent snuffbox or wrist AV fistula at Betsi Cadwaladr University Health Board were studied.

Data collection

We retrieved the data of AV fistula patients who underwent snuffbox and wrist AV fistula at Betsi Cadwaladr University Health Board between January 2013 and January 2023. The clinical evaluation included factors such as the patient’s age, gender, site of fistula creation, smoking history, comorbidities, fistula failure, primary, assisted primary, and secondary patency.

Surgical technique

The fistula was established using local anaesthesia (0.5% bupivacaine and 0.5% lidocaine). An initial antibiotic was administered. A 2-3 cm-long skin incision was made across the first interosseous gap or the wrist. This provides adequate exposure to both the artery and vein. Continuous polypropylene sutures (6/0 or 7/0 proline) were used to create an end-to-side anastomosis between the cephalic vein and radial artery. The wound was closed in layers, and a palpable thrill was palpated to indicate success.

Follow-up

The AV fistula was assessed by Doppler ultrasound after six weeks of creation and considered mature if the flow was more than 400 mL/minute. The patient underwent a surveillance Doppler ultrasound scan every six months.

Definition

Fistula failure was considered when the fistula maturation failed or the ability to use the fistula for dialysis failed.

Data analysis

The data collected for this study was analysed using SPSS version 21 software (IBM Corp., Armonk, NY, USA), including data entry, cleaning, and analysis. Descriptive statistics were utilised to present the frequency tables with corresponding percentages. The means and standard deviations were also reported. A bivariate analysis was conducted to assess the associations between the outcome variables and other relevant influencing factors. The statistical tests employed were the chi-square test for categorical variables and the t-test for quantitative variables. Kaplan-Meier curves were used for survival analysis to compare the two groups using the log-rank test. A significance level of 0.05 or less was considered statistically significant, indicating a substantial relationship between the variables.

## Results

In a period of 10 years, 179 patients were included. Overall, 102 (57%) were male and 77 (43%) were female, with a male-to-female ratio of 1.3:1. The mean age was 68.7 ± 13.1 years. Regarding the comorbidities, hypertension and diabetes were recorded for most patients, while the other comorbidities were less frequent (Table 1).

**Table 1:** Patient demographics and comorbidities. PVD = peripheral vascular disease

		Snuffbox	Wrist	P-value
Gender	Male	17	85	0.007
	Female	26	51	
Haemodialysis	Yes	19	66	0.374
	No	24	70	
Hypertension	Yes	24	99	0.018
	No	22	34	
Diabetes	Yes	31	94	0.021
	No	12	42	
Cardiac diseases	Yes	22	49	0.061
	No	21	87	
Smoking	Yes	18	71	0.166
	No	27	63	
PVD	Yes	8	22	0.473
	No	43	106	

The wrist radiocephalic fistula was the dominant type of surgery done at 76% (n = 136), while the snuffbox radiocephalic fistula was less common at 24% (n = 43) (Table 2). Overall, 23 patients had previous AV fistulas; 11 had wrist AV fistulas, seven had snuffbox, three had brachiocephalic, and two had brachiobasilic fistulas (Table 2).

**Table 2:** AV fistula characteristics and outcome. AV = arteriovenous; BCF = brachiocephalic fistula; BBF = brachiobasilic fistula

		Snuffbox	Wrist	P-value
Side	Right	10	45	0.152
	Left	33	91	
Previous fistula	Yes	5	18	0.502
	No	39	117	
Site of the previous fistula	Snuffbox	0	7	0.313
	Wrist	4	7	
	BCF	0	3	
	BBF	0	2	
	Not applicable	38	117	
Central vein stenosis	Yes	0	2	0.000
	No	43	134	
Mortality	Yes	10	81	0.000
	No	33	55	
Complications	Bleeding	0	13	0.10
	infection	2	25	
	Pseudoaneurysm	0	5	
	Blockage	6	21	
	Stenosis	20	40	
	No complications	21	26	
Fistula failure	Yes	20	47	0.127
	No	29	83	
Intervention for fistula salvage	Surgery	0	8	0.039
	Endovascular	23	47	
	No intervention	20	81	

In total, 124 (69.3%) patients had AV fistulas created on the left side, and only 55 (30.7%) patients had AV fistulas created on the right side. Overall, 85 (47.5%) patients who were already on hemodialysis had AV fistula; however, 94 patients had AV fistula before they started dialysis. Only two (1.1%) patients were known to have central vein stenosis (Table 2). Overall, 91 (50.8%) patients died. Regarding the complications, the most common were stenosis (33.5%), followed by blockage, infection, bleeding, and pseudoaneurysm.

Most patients had a successful AV fistula (105, 58.7%), in contrast to 67 patients whose fistulas failed. There was a significant relationship between fistula failure and complications (p = 0.000) (Table 3). There was no significant effect of the fistula site, hypertension, diabetes, cardiac diseases, smoking, peripheral vascular disease, or central vein stenosis on the failure of AV fistula (p = 0.127, 0.534, 0.510, 0.397, 0.017, 0.68, and 0.371, respectively).

**Table 3:** Factors affecting fistula failure. P-value = 0.000. This table shows the factors that were significantly related to fistula failure.

		Bleeding	Infection	Pseudoaneurysm	Blockage	Stenosis	No complications
Fistula failure	Yes	7	6	0	27	21	21
	No	6	20	5	0	39	39
Total		13	26	5	27	60	60

When performing the regression analysis to identify the independent diameter for fistula failure, it was 1.8 mm for the vein and 1.5 mm for the artery (p = 0.003 and 0.004, respectively).

**Figure 1:**
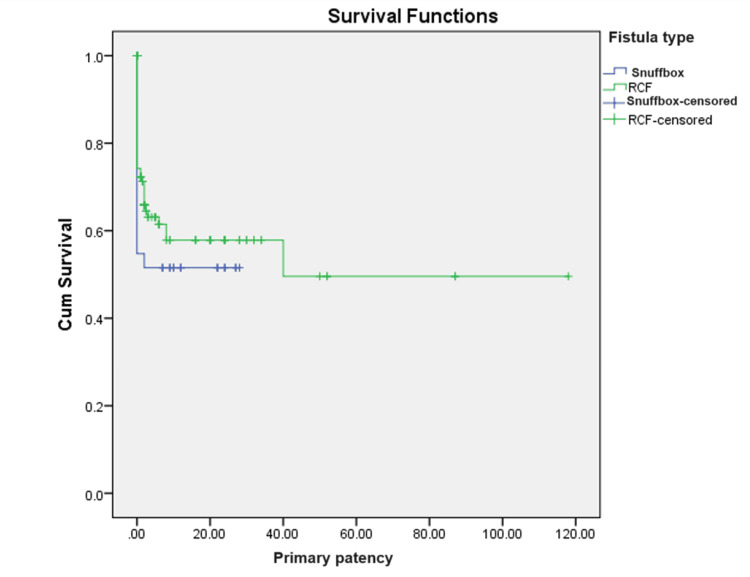
Kaplan-Meier survival curves for primary patency. P-value = 0.433. RCF = wrist radiocephalic fistula

**Figure 2:**
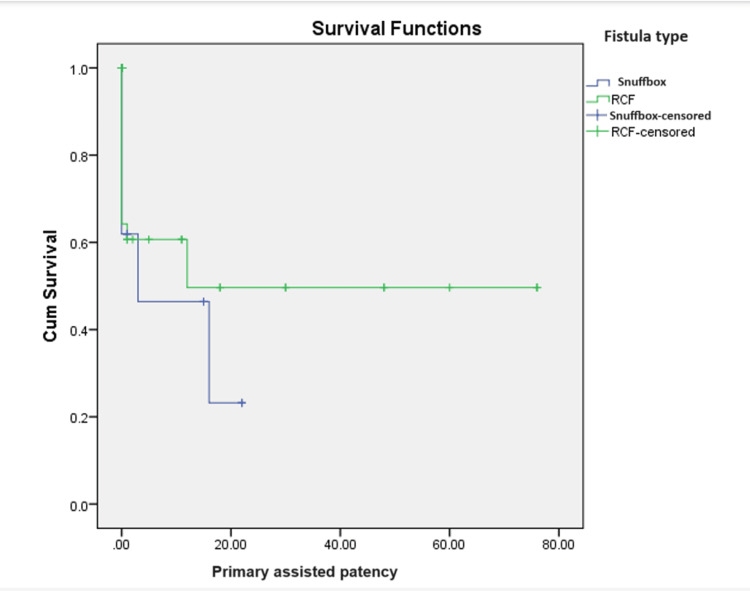
Kaplan-Meier survival curves for primary assisted patency. P-value = 0.433. RCF = wrist radiocephalic fistula

**Figure 3:**
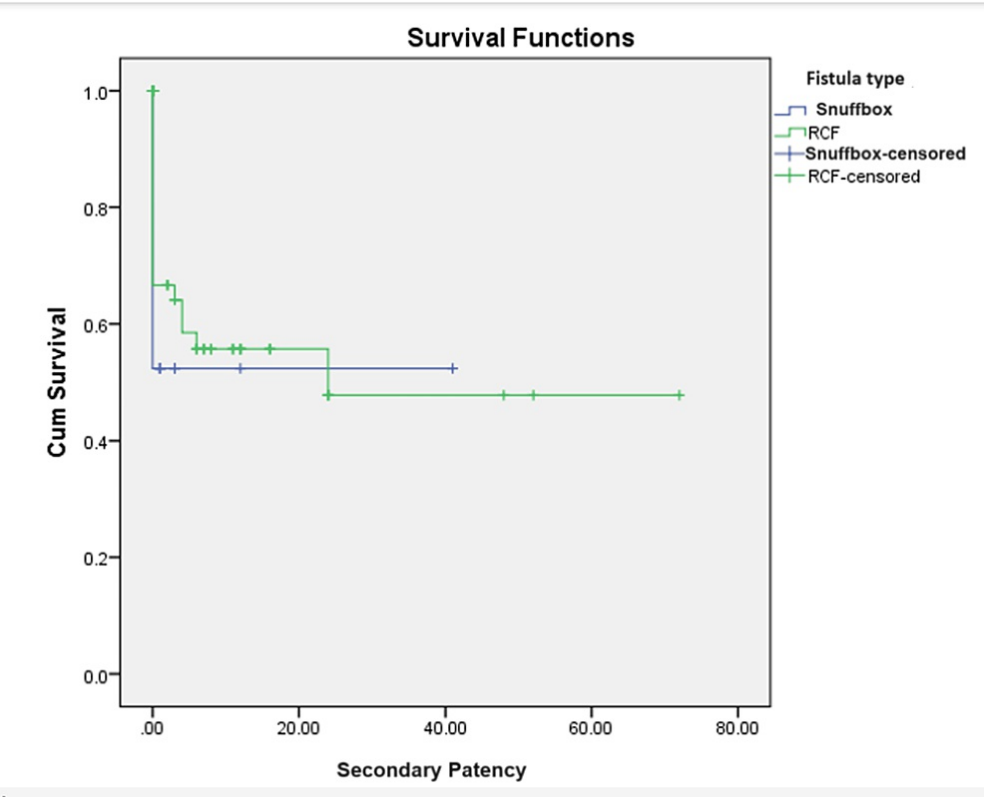
Kaplan-Meier survival curves for secondary patency. P-value = 0.153. RCF = wrist radiocephalic fistula

A total of 78 patients underwent a procedure to salvage AVF. Eight underwent surgery and 70 underwent an endovascular intervention. The vein diameter by duplex ultrasound ranged from 1.5 to 5 mm, with a mean of 2.99 ± 0.6 mm. The artery diameter ranged from 1 to 4.4 mm, with a mean of 2.5 ± 0.5 mm. The mean weeks for fistula maturation were 6 ± 7 weeks. On comparing the means of maturation among the two groups, there was a significant difference in maturation time (Table 4). The primary patency ranged from 1 to 118 months, with a mean of 8.4 ± 16.4 months. The mean primary assisted patency ranged from 1 to 76 months, with a mean of 3.3 ± 11.5 months. The secondary patency ranged from 1 month to 72 months, with a mean of 2.9 ± 9 months, as shown in the Kaplan-Meier survival curves (Figures 1-3, respectively). On comparing the mean between the two groups, it did not show any significant difference between primary, primary assisted, or secondary patency (p = 0.052, 0.233, and 0.065, respectively).

**Table 4:** Comparison of the maturation mean. P-value = 0.033. RCF = wrist radiocephalic fistula

				Confidence interval	
	Fistula type	Mean	Standard deviation	Lower	Upper
Maturation time between groups	Snuffbox	7.1	6.8	1.9	3.2
	RCF	6.5	7.8	1.8	3.1

## Discussion

Our study concluded that there were similar outcomes between snuffbox AVFs and wrist AVFs concerning maturation, postoperative complications, patency, and failure. According to this result, the more proximal radial artery can be retained without compromising outcomes, resulting in snuffbox AVF as a viable option for wrist AVF for distal access.

Our study showed that only 43 SBAVFs were created during the study duration in our centre in comparison to wrist AVFs. This may be due to the unfamiliarity of most surgeons with this particular type of access or the anatomic site, as well as the comfort of the surgeon with the procedure. Moreover, these factors might also affect the outcome, as reported in previous studies [6-10].

There was no significant impact of the comorbidities, smoking, and peripheral arterial disease on fistula maturation or failure. Horimi et al. examined the impact of diabetes on outcomes [11]. Diabetics generally have significantly reduced fistula patency and flow rates. However, no risk-adjusted analysis was done. Our analysis did not turn out to be a significant predictor.

Previous studies attempted to determine the typical characteristics of vessels necessary for a successful AVF; however, they obtained various results. Kim et al. determined that the lack of development in RCAVF is associated with preexisting intimal hyperplasia in the radial artery, a condition frequently found in patients with renal disease. Lauvao et al. showed that vein diameter is a significant predictor of fistula success [12-17].

In their study, Hull et al. established the criteria that the vessels (artery and vein) used in the AVF creation should have a diameter greater than 2 mm, which is consistent with the findings of our investigation [18]. Early failure of the distal AVF has been observed in many studies [6,11,13]. In our study, we did not examine the time when the failure occurred because of a lack of this information in the records and because the data were collected retrospectively.

In our study, there was no significant difference in terms of primary, assisted primary, and secondary patency rates. Therefore, the SBAVF is a good option for starting [10,13,19]. Some studies have mentioned the early high patency rate of SBAVF, which aligns with our earlier results. However, most studies have not addressed the long-term patency rate [7,19]. Simoni et al. compared the patency rates of SBAVF to those of wrist AVF and found that the results were comparable, with SBAVF patency rates of 77.3% at one year, 36.3% at five years, and 18.9% at 10 years compared to wrist AVF patency rates of 75.5% at one year, 54.5% at five years, and 30.7% at 10 years [10]. There was no significant difference among the two groups in terms of outcome, which is aligned with some previous studies [10,17].

Our study has a few limitations. We retrospectively collected data. The reason why a particular access type was chosen remains unknown. It is a monocentric, descriptive, comparative analysis between two heterogeneous small groups. Indeed, there was a large difference between the total number of patients in the two groups.

## Conclusions

There was no significant difference between the SBAVF and the wrist AVF. Regarding outcome, patency, and rate of complications, the SBAVF fistula is a viable and realistic option for patients undergoing hemodialysis. If the wrist AVF is performed first, the opportunity to create the SBAVF is lost. To provide vascular access, we recommend using SBAVF, especially for younger patients who do not have any comorbidities.

## References

[1] Mokhtari S, Besancenot A, Beaumont M, Leroux F, Rinckenbach S, Salomon Du Mont L (2022). Snuff-box versus wrist radiocephalic arteriovenous fistulas for hemodialysis: maturation tend and its affecting factors. Ann Vasc Surg.

[2] Moeller S, Gioberge S, Brown G (2002). ESRD patients in 2001: global overview of patients, treatment modalities and development trends. Nephrol Dial Transplant.

[3] Grassmann A, Gioberge S, Moeller S, Brown G (2005). ESRD patients in 2004: global overview of patient numbers, treatment modalities and associated trends. Nephrol Dial Transplant.

[4] Wilmink T (2018). Vascular access: clinical practice guidelines of the European Society for Vascular Surgery. Eur J Vasc Endovasc Surg.

[5] Rassat JP, Moskovtchenko Moskovtchenko, Perrin J, Traeger J (1969). [Artero-venous fistula in the anatomical snuff-box]. J Urol Nephrol (Paris).

[6] Lee T (2017). Fistula first initiative: historical impact on vascular access practice patterns and influence on future vascular access care. Cardiovasc Eng Technol.

[7] Wolowczyk L, Williams AJ, Donovan KL, Gibbons CP (2000). The snuffbox arteriovenous fistula for vascular access. Eur J Vasc Endovasc Surg.

[8] Twine CP, Haidermota M, Woolgar JD, Gibbons CP, Davies CG (2012). A scoring system (DISTAL) for predicting failure of snuffbox arteriovenous fistulas. Eur J Vasc Endovasc Surg.

[9] Letachowicz K, Gołębiowski T, Kusztal M, Letachowicz W, Weyde W, Klinger M (2016). The snuffbox fistula should be preferred over the wrist arteriovenous fistula. J Vasc Surg.

[10] Simoni G, Bonalumi U, Civalleri D, Decian F, Bartoli FG (1994). End-to-end arteriovenous fistula for chronic haemodialysis: 11 years' experience. Cardiovascular Surgery.

[11] Horimi H, Kusano E, Hasegawa T, Fuse K, Asano Y (1996). Clinical experience with an anatomic snuff box arteriovenous fistula in hemodialysis patients. ASAIO J.

[12] Beigi AA, Masoudpour H, Alavi M (2009). The effect of ligation of the distal vein in snuff-box arteriovenous fistula. Saudi J Kidney Dis Transpl.

[13] Bonalumi U, Civalleri D, Rovida S, Adami GF, Gianetta E, Griffanti-Bartoli F (1982). Nine years' experience with end-to-end arteriovenous fistula at the 'anatomical snuffbox' for maintenance haemodialysis. Br J Surg.

[14] Khavanin Zadeh M, Gholipour F, Naderpour Z, Porfakharan M (2012). Relationship between vessel diameter and time to maturation of arteriovenous fistula for hemodialysis access. Int J Nephrol.

[15] Kim YO, Song HC, Yoon SA (2003). Preexisting intimal hyperplasia of radial artery is associated with early failure of radiocephalic arteriovenous fistula in hemodialysis patients. Am J Kidney Dis.

[16] Lauvao LS, Ihnat DM, Goshima KR, Chavez L, Gruessner AC, Mills JL Sr (2009). Vein diameter is the major predictor of fistula maturation. J Vasc Surg.

[17] Hull JE, Kinsey EN, Bishop WL (2013). Mapping of the snuffbox and cubital vessels for percutaneous arterial venous fistula (pAVF) in dialysis patients. J Vasc Access.

[18] Siracuse JJ, Cheng TW, Arinze NV (2019). Snuffbox arteriovenous fistulas have similar outcomes and patency as wrist arteriovenous fistulas. J Vasc Surg.

[19] Lee JK, Sara TT (2000). Experience in snuffbox arteriovenous fistulae for hemodialysis. Med J Malaysia.

